# The human microbiota: a double-edged sword against the ‘Sword of Damocles’ in PDAC diagnosis and therapy

**DOI:** 10.3389/fonc.2025.1519277

**Published:** 2025-06-26

**Authors:** Maria Sindaco, Leonardo Mercanti, Valentina Puca, Mariangela Mazzone, Maria Carmela Di Marcantonio, Raffaella Muraro, Michele Fiordaliso, Gabriella Mincione

**Affiliations:** ^1^ Department of Innovative Technologies in Medicine & Dentistry, University “G. d’Annunzio” of Chieti-Pescara, Chieti, Italy; ^2^ Department of Pharmacy, University “G. d’Annunzio” of Chieti-Pescara, Chieti, Italy; ^3^ Department of Medicine and Ageing Sciences, University “G. d’Annunzio” of Chieti–Pescara, Chieti, Italy

**Keywords:** microbiota, PDAC, TME, IPMN, immunotherapy, FMT, bacteriotherapy, viral vectors

## Abstract

Pancreatic Ductal Adenocarcinoma (PDAC) is currently a major oncological threat given the very low 5-year survival rates of 8-9%. The tumor itself is intertwined with its surrounding tissue in a peculiar tumoral microenvironment (TME) which contributes to resistance against the host immune system and traditional clinical treatments, such as chemotherapy. One of the components of TME is the microbiota, which mainly includes the bacterial species identified in the tumor tissue at various stages. Current literature highlights an active role of the microbiota in tumorigenesis, progression, metastasis, and chemotherapy response in PDAC patients. This review gathered the most recent findings about microbial composition in PDAC patients, along with the effects of intra and extra-tumoral (GI and oral) microbial species on the TME and immune system, their role in tumor progression and immuno-modulation. This paper provides an insight on the potential use of microbes as diagnostic and prognostic markers, and as an additional therapeutic strategy. The study of microbiota offer new ways to slow down carcinogenesis, modulate the immune response, and even serve as an early diagnostic tool in the absence of specific serum markers. In the current review we will offer an inquiry on these potential roles. We sorted out the most recent literature with a comprehensive and critical approach, sourcing papers from PubMed. We exclusively opted for papers that were published in the last 5 years on journals with IF≥4, with a focus on the impact of intra-tumoral microbiome on the natural history of PDAC, from pre-tumoral lesions to metastasis.

## 1 Introduction

Pancreatic ductal adenocarcinoma (PDAC) is currently one of the most formidable oncological challenges. Placing itself at number 7 in the most common oncological causes of death worldwide, with a slight preference for males over females (1, 2). PDAC is associated with a 5-year survival rate of only 8-9%, reflecting its highly aggressive nature (3). In most cases, a diagnosis of PDAC occurs at an advanced stage, where surgery may not be a feasible treatment approach. This mostly happens because of the paucisymptomatic nature of PDAC, combined with a lack of specific early diagnostic markers and tools. The most relatively reliable marker, CA 19-9, remains still underperforming with low specificity and sensibility (1). Risk factors for PDAC have yet to be confirmed, although literature indicates that environmental and modifiable factors, such as an unbalanced diet and high BMI, may pose a higher threat to cancer development than genetic and hereditary components. The predominant advanced age of diagnosis for PDAC patients (70–80 years) suggests a stochastic model for cancer development, rather than a genetics-driven development (the latter is usually associated with earlier development). Nonetheless, PDAC among blood relatives poses a higher risk for the development of new cases (1).

A key feature of PDAC is its close association with the surrounding tissue, which forms a unique tumor microenvironment (TME) with a significant role in resistance to both the immune system and conventional treatments like chemotherapy (4).

The TME could be defined as a borderline-pathological stroma which lays at the interface between the tumoral mass and the surrounding physiological pancreatic tissue.

The main component of the PDAC TME is the extracellular matrix (ECM), a firm acellular and fibrotic three-dimensional network, which is composed for the most part of type I, II and XV collagens, fibronectin, tenascin, deposed by cancer-associated-fibroblasts (CAFs) post stimulation by PC cell (5, 6).

The desmoplastic matrix plays a pivotal role in determining the marked chemoresistance of PDAC, as it generates high interstitial pressure, with a constricting effect on blood vessels (7, 8). It would seem that GAGs make a remarkable contribution in the aforementioned process, by binding water molecule and expanding in volume (7).

The packed desmoplastic stroma is densely populated by heterogeneous cell populations, which are responsible for PDAC progression.

Pancreatic Stellate Cells (PSCs) are remarkably abundant around pancreatic acini, as they constitute about 5-7% of all the cells in the pancreatic parenchyma (5). Physiologically they serve as depositories for Vitamin A (5). Nevertheless, in the context of pancreatic chronic inflammation, PSCs are activated by proinflammatory cytokines and growth factors (e.g., IL-1β, IL-6, TNF-α, TGF-β1), resulting in their transformation into Cancer Associated Fibroblasts (CAFs) (9). CAFs are responsible for the secretion of the components of PDAC ECM (5, 8, 10). They also seem to be able to assume myocyte-like and proinflammatory roles (11, 12). The increasing interest in their functions and their structural heterogeneity by the scientific community resulted in various molecular sub-classifications (11–13).

Moreover, both proinflammatory (e.g., M0 macrophages, neutrophils and memory B cells) and immuno-modulating (e.g., CD8^+^ and CD4^+^ T lymphocytes, naïve B cells, monocytes, plasma cells, and activated mast cells) leukocyte populations abundantly reside into PDAC TME, with the latter being more abundant in tumor samples from patients with worse prognosis (14).

Another important component of the TME is the microbiota, consisting primarily of bacterial species that colonize the tumor tissue at different stages of the disease (15).

Formerly considered sterile, healthy pancreas is currently known to be infiltrated by various microbial species (16). While the ways bacteria reach the pancreas are still worthy of further investigation, it would seem that some bacteria species are able to translocate from the gastrointestinal tract to the pancreatic parenchyma (17).

Several studies highlighted the variability in microbial composition between specimens of healthy pancreas and PDAC (3, 4, 15, 18, 19). Indeed, samples from PDAC patients with different prognosis revealed remarkable dissimilarity in the abundance of certain microbial species, confirming their active role in determining PDAC progression and natural history (20–22). Microbiota imbalances, termed dysbiosis, can trigger chronic inflammation, which in turn favor tumorigenesis.

Recent studies have uncovered the potential of microbiome alterations to support early PDAC tumorigenesis, as it is demonstrated by the precocious presence of dysbiosis in pre-tumoral lesions (23, 24). Bacterial and viral species promote pancreatic neoplastic degeneration both through a direct interfering effect on pancreatic cells genome and as major promoters of oxidative stress (24, 25).

Moreover, dysbiosis is involved in the subsequent stages of PDAC progression, as microbial species interact with PC cells and immune cells in several various ways: these include favoring PDAC cells epithelial mesenchymal transition (26), secreting both pro-tumorigenic and cancer-unfavoring metabolites (27, 28) and exerting immune-modulating functions (24, 29–32).

Notably, microbiota from other body regions, such as the oral cavity and gastrointestinal tract, has also been implicated in PDAC (33, 34). One of the clearest examples of this phenomenon is *Helicobacter pylori* - mediated dysplasia in the stomach mucosa, which can potentially degenerate into gastric MALT-lymphoma (35); another case of microbiota-associated neoplastic disorder is the relationship between *P. gingivalis* and oral squamous cell carcinoma, with growing evidence of a cause-and-effect sequence in the presence of this bacterial strain (36).

These findings have raised a great interest in the microbiota as a target for new strategies to slow tumor progression, modulate immune responses, and even serve as an early diagnostic tool in the absence of specific serum markers.

This manuscript is the result of a careful evaluation of the potential roles of the microbiota, providing a comprehensive and critical analysis of recent literature and summarizing existing studies to form a cohesive dissertation. The goal of this review is to highlight emerging diagnostic and therapeutic strategies that could reshape the approach to PDAC management.

## 2 Review strategies and literature included

**Figure 1:**
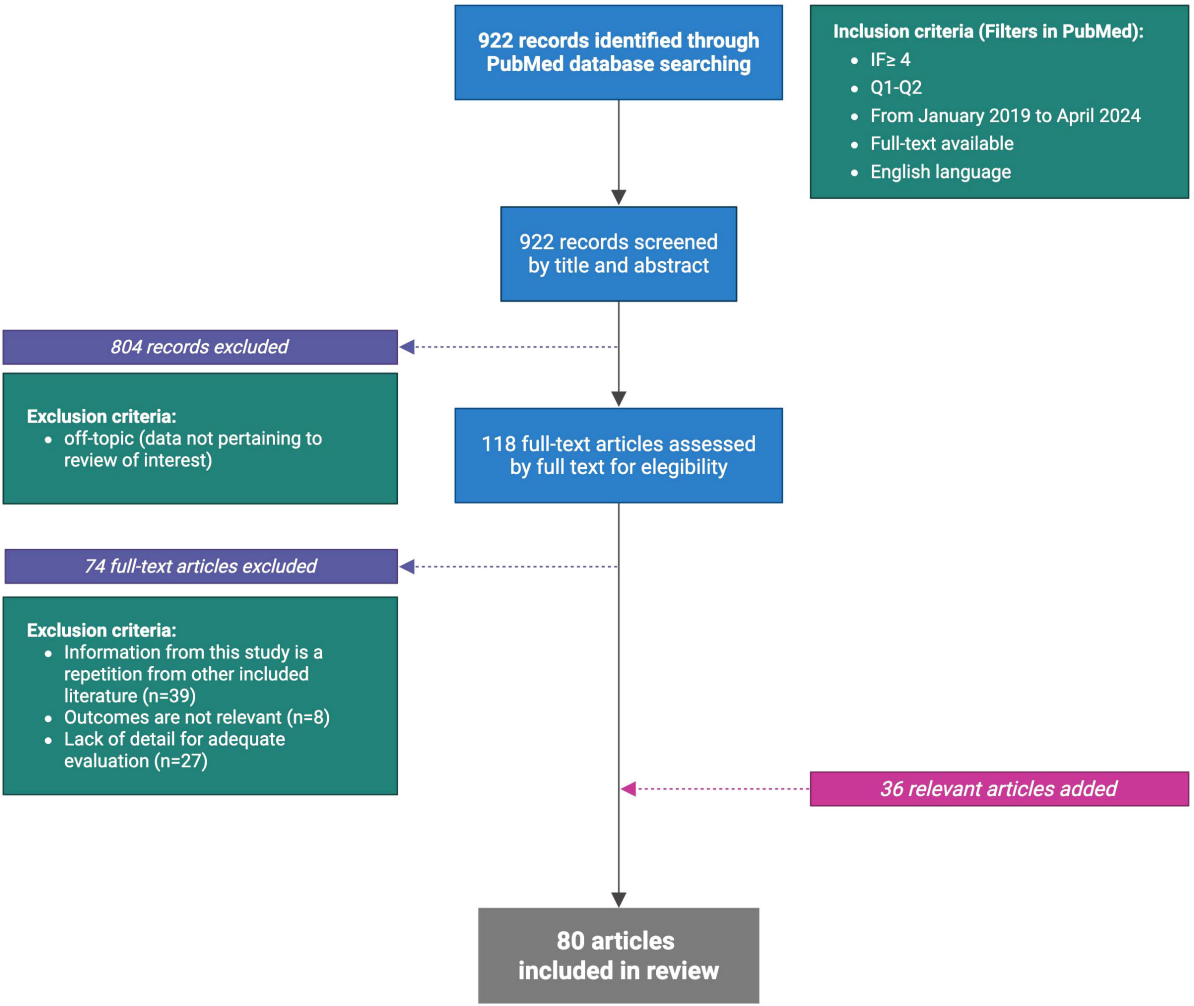
Flow chart of literature selection (Created in BioRender.com).

For this review, the PubMed database was used for the articles search. The keywords were: “PDAC and microbiota”, “PDAC and microbiome”, “PDAC and microbiota or PDAC and microbiome”, “PDAC microbiota and immune system”, “PDAC microbiome and immune system”, “PDAC and bacteria”, “PDAC and virus”, “PDAC and fungi”, “PDAC and Fecal Microbiota Transplant (FMT)”. Records in the English language, with full-text available, that were published (or accepted for publication) between January 2019 and April 2024, published in journals with impact factors ≥4 and quartiles Q1 or Q2 were included, except for 25 articles which did not meet all the search criteria but proved essential for the writing of the review. The primary search provided 922 records. The first step excluded all articles with data not pertaining to review of interest (804 removed). The following step excluded all full-text articles with the same information of other included literature (39 removed) or had not relevant outcomes (8 removed) or presented lack of detail for adequate evaluation (27 removed). Further, consultation of references from the gathered articles provided 11 additional articles not included in the initial screening but eligible within the search criteria. After applying these criteria, 80 articles provided the core literature for the current review (**Figure 1**).

## 3 Microbial asset in healthy pancreas and microbial species in PDAC TME

Microbiota plays an essential role in maintaining homeostasis in the human body, and an imbalance of the microbiota, a state known as dysbiosis, can contribute to the pathogenesis of many diseases (37). It is well recognized that most microorganisms reside in the gastrointestinal tract, but part of them can be found in other districts of the human body such as skin, oral cavity and genitourinary tract. For many years, the pancreas was considered a sterile organ, but recent studies demonstrated the existence of microorganisms in both normal and tumoral pancreatic tissues (15, 16, 20, 38). The way bacteria enter the pancreas is still controversial, and could include some mechanisms, such as oral route and by translocation from the lower gastrointestinal tract. In the first study which started to investigate how microbes reach the pancreas, the authors demonstrated that labeled bacteria or fungi administered to mice via the oral route can reach the pancreas via the duodenum within a couple of hours (17). The source of bacteria in the pancreatic tissue probably is due to the migration of bacteria from the oral cavity and gastrointestinal tract to the pancreas through the common pancreatic duct. Furthermore, pathogens could disseminate from the colon, gallbladder and kidneys to the pancreas.

The presence of microorganisms in human tumors was first detected more than 100 years ago, but the identification of tumor microbiota still remains a challenge because of its low biomass as well as the possibility of tumor sample contamination (16). For these reasons, publications describing the healthy pancreatic microbiota are very rare. Most studies use tumor-adjacent normal tissue or pancreatic tissue from benign diseases as controls. Geller et al. first detected bacterial 16S rDNA in healthy pancreas samples from organ donors by Real-time quantitative polymerase chain reaction (q-PCR). However, they only detected bacterial DNA in 15% of these control samples as opposed to 76% in PDAC samples (4). Thomas et al. did not show any significant differences between healthy pancreatic and tumor tissue, although they observed higher abundances of *Acinetobacter*, *Enterobacter*, and *Pseudomonas* genera in normal pancreas (18). Differently, Pushalkar et al. found that *Chlamydiales* and *Brevibacterium* showed higher relative abundance in normal human pancreatic tissue as compared with PDAC tissues (19).

The following selected articles highlight how distinct microbial communities within pancreatic tumor tissue can potentially influence PDAC occurrence and development as well as response and prognosis of pancreatic cancer to treatment (**Table 1**).

**Table 1:** Overview on microorganisms identified in pancreatic cancer and in pancreatic adjacent normal tissue, described in recent studies.

Samples	Sequencing Method	Microbiome	Conclusions	Reference
Fresh human PDAC samples; PDX mice samples	18S rRNA sequencing and mycobiome analysis	PDAC tumors are infiltrated mostly by fungal genera such as *Alternaria* and *Malassezia*	Intratumor fungi facilitate the secretion of IL-33 from PDAC mice cells and accelerate PDAC mice tumor growth.	(41)
Mice and human fecal and pancreatic tissue samples	Real-time qPCR and FISH using 28S rRNA gene probe labeled with the 5’Cy3 fluorophore; 18S rRNA gene sequencing	Mice and human PDAC samples presented a ~3000-fold increase in fungi compared to normal pancreas. Enrichment of *Malassezia* in both mice and human PDAC samples, that accelerates oncogenesis.	Pathogenic fungi promote PDAC progression by activating the complement system via MBL activation.	(40)
Normal pancreatic tissue; PDAC and IPMN tissues.	16S rRNA gene/amplicon sequencing	Phylum level: Proteobacteria (82.3% normal, 77.6% IPMN, 81.1% PDAC), Bacteriodetes (11.4% normal, 11.1% IPMN, 12.8% PDAC), Actinobacteria (0.8% normal, 1.2% IPMN, 1.5% PDAC), and Firmicutes (0.7% normal, 2.3% IPMN, 0.3% PDAC) Genus level: *Pseudoalteromonas* (24.7% normal, 21.9% IPMN, 14.6% PDAC), *Pseudomonas* (14.0% normal, 16.5% IPMN, 13.5% PDAC), Vibrio (8.9% normal, 5.1% IPMN, 4.9% PDAC), and *Sediminibacterium* (5.6% normal, 9.3% IPMN, 7.9% PDAC).	No statistically significant differences in bacteria composition or diversity between normal pancreas, IPMN, or PDAC, probably due to low presence of microbial DNA.	(16)
Tumor samples resected from LTS and STS PDAC survivors	16S rRNA amplicon sequencing	LTS samples exhibited higher abundances of *Sphingomonas*, *Megasphaera*, *Bradyrhizobium*, hgcI_clade, *Desulfovibrio*, *Flavobacterium*, *Enhydrobacter*, and *Megamonas*, STS samples exhibited higher abundances of *Clostridium_sensu stricto* 1, *Actinomyces*, *Porphyromonas*, *Aggregatibacter*, and *Neisseria*	Specific intratumor microbiome can enhance the anti-tumor effect in the host, laying the foundation for further elucidating the underlying detailed mechanism.	(3)
Archived PDAC tissues along with matched adjacent normal tissue samples.	16S rRNA (amplicon) sequencing	Phylum level: in PDAC, Actinobacteroita (39.2%), Proteobacteria (31.3%), and Bacteroidota (14.4%) were high; in adjacent normal tissues Firmicutes (41.1%), Actinobacteroita (23.7%), Proteobacteria (18.7%), and Cyanobacteria (11.6%). Genus level: *Streptomyces*, *Cloacibacterium*, and *Corynebacterium* abundance in PDAC; in adjacent normal tissues, *Acholeplasma* was the most predominant, followed by *Streptomyces.* Genera across tumor stages: *Gemella* (prevalence 7.69%), *Lactobacillus* (prevalence 7.69%), *Microlunatus* (prevalence 7.69%), and *Bacillus* (prevalence 15.38%) exclusively appeared in G3 stage samples. *Cetobacterium*, *Enterococcus*, and *Fusobacterium* detected in a few samples of the G2 and G3 groups but absent in G1 stage samples.Higher abundances of *Delftia* and *Staphylococcus* compared in the G1 compared with G2 and G3/G4 stages . The relative abundance and prevalence of *Delftia* were 31.0 and 100%, respectively, in G1 samples, while *Cloacibacterium* (relative abundance 15.7% and prevalence 15.38%) and *Actinobacter* (relative abundance 14.9% and prevalence 66.6%) were abundant in G2 and G3, respectively.	PDAC lesions harbor relatively different microbioma compared with their normal tumor adjacent tissues. The relative abundance of *Delftia* in G1, suggests its potential role in tumor development and progression paving the way for further exploration as a potential molecular marker for diagnosis and prognosis of PDAC.	(15)
Archived PDAC resected tissues of STS and LTS patients ().	16S rRNA gene (amplicon) sequencing	Class level: predominance of *Alphaproteobacteria Sphingobacteria*, and *Flavobacteria* in LTS PDAC. *Clostridia* and *Bacteroidea* in STS PDAC. Genus level: abundance of *Proteobacteria* (*Pseudoxanthomonas*) and *Actinobacteria* (*Saccharopolyspora* and *Streptomyces*) in LTS; no abundance in STS.	The distinct tumor microbiome from PDAC LTS can be used to predict tumor survival in humans, and transfer of LTS gut microbiomes can alter the tumor microbiome and tumor growth in mouse models. The presence and abundance of Saccharopolyspora, Pseudoxanthomonas and Streptomyces, together with the presence of *Bacillus clausii*, could influence and predict long-term survivorship in PDAC patients.	(22)
101 samples of patients with PDAC in tumor and in surrounding normal pancreatic tissue.	DNA extraction and PCR	68% of all patients were HHV-6 DNA positive in any of the samples, 49% were positive in tumor tissue. Specimens of just one patient were HHV-6A DNA positive, all others were positive for HHV-6B.	HHV-6 DNA-positivity of PDAC tissue is probably not due to the infection of pancreatic cells by HHV-6, but to the migration of HHV-6 positive immune cells into the pancreas. No direct evidence for HHV-6 as a causative agent.	(38)
582 samples from PDAC tissues across four datasets selected which contain 214, 191, 30, and 147 samples, respectively.	Four PDAC datasets with mRNA sequencing data	Identification of 54 core microbiomes across the four datasets, among which the genera *Kocuria*, *Streptococcus*, *Bacillus*, *Lactobacillus*, *Ralstonia*, *Staphylococcus*, *Acinetobacter*, *Pseudomonas* were shared. In mice PDX samples, *Staphylococcus*, *Ralstonia*, *Moraxella* and *Cloacibacterium* showed higher abundance, whereas *Corynebacterium*, *Streptococcus*, *Bacillus*, *Pseudomonas*, *Acinetobacter*, *Salmonella*, *Massilia*, *Lactobacillus*, *Halomonas*, *Pantoea*, and *Clostridium* were highly abundant in human tumor tissue samples.	The results play important guiding roles in the follow-up studies of tumor microbiome, such as the role of intra-tumor bacteria on anti-tumor immunity.	(39)
105 tumors and 101 paired peritumoral tissues surgically resected from PDAC patients. Further analysis of tumor and paired peritumoral tissues of LTS and STS groups.	16S rRNA gene/amplicon sequencing	Phylum level: predominance of Firmicutes in PDAC tissues; Proteobacteria, (gammaproteobacteria) in peritumoral tissues; Genus level: abundance of *Exiguobacterium* and *Stenotrophomonas* in PDAC; *Lysobacter* in peritumoral tissue; Order level: Bacillales in PDAC; Betaproteobacteriales in peritumoral tissue.	Similarities and differences in microbial compositions between tumor tissues and their adjacent peritumoral tissues. *Bacillus* could exert inhibitory effects on tumor progression of PDAC, whereas *Exiguobacterium* could play a role in facilitating the advancement of PDAC. Specific bacteria can be identified within both tumor and peritumoral tissues and could serve as prognostic biomarkers for patients with PDAC.	(20)

PDX, patient-derived xenografts; MBL, mannose-binding lectin; IPMN, intraductal papillary mucinous neoplasm; LTS, long-term PDAC survivors; STS, short-term PDAC survivors.

Huang and co-authors, confirm that the relative abundance of bacteria in the tumor tissue is higher than that in paracancerous tissue (3). The authors divided PDAC resected tissues in long-term and short-term PDAC survivors (>5-years overall survival, median survival 10.14 years, called LTS; overall survival less than 5 years, median survival 1.62 years, called STS, respectively) (**Table 2**). LTS samples showed abundances of *Sphingomonas*, *Megasphaera*, *Bradyrhizobium*, *hgcI_clade*, *Desulfovibrio*, *Flavobacterium*, *Enhydrobacter*, and *Megamonas*, while STS samples exhibited higher abundances of *Clostridium_sensu stricto* 1, *Actinomyces*, *Porphyromonas*, *Aggregatibacter*, and *Neisseria*. *Clostridium*, which is considered a genus of opportunistic pathogens (21), was higher in most STS samples than in LTS samples. The authors decided to go beyond the metagenomic results to evaluate if the administration of bacterial species identified in long-term survivors could influence the tumor growth. They administrated *Megasphaera*, one of the differential bacteria, in LTS samples in mice and report that it induced a better tumor growth inhibition effect when combined with the immune checkpoint inhibitor anti-programmed cell death-1 (anti-PD-1) treatment in mice with 4T1 tumor.

**Table 2:** Comparison of the abundance of microbial species between Long Term (LTS) *versus* Short Term PDAC Survivors (STS).

LTS Microbiome	STS Microbiome	Reference
Genus level: higher abundances of *Clostridium_sensu stricto* 1, *Actinomyces*, *Porphyromonas*, *Aggregatibacter*, and *Neisseria*	Genus level: higher abundances of *Sphingomonas*, *Megasphaera*, *Bradyrhizobium*, hgcI_clade, *Desulfovibrio*, *Flavobacterium*, *Enhydrobacter*, and *Megamonas*	(3)
Class level: predominance of *Alphaproteobacteria Sphingobacteria*, and *Flavobacteria* Genus level: abundance of *Proteobacteria* (*Pseudoxanthomonas*) and *Actinobacteria* (*Saccharopolyspora* and *Streptomyces*)	Class level: *Clostridia* and *Bacteroidea* Genus level: no abundance	(22)
Genus level: higher relative abundance of *Bacillus*, lower abundance of *Exiguobacterium*	Phylum level: a dominance of Deinococcus_Thermus Class level: a dominance of Deinococci	(20)

Khan and co-authors, carried out a comparison between the microbiome of PDAC tissue and that of adjacent normal tissue. Within both tissue types, the main phyla detected were Proteobacteria, Actinobacteroita, Firmicutes (now renamed to Bacillota), Bacteroidota, and Cyanobacteria. There were variations in the relative abundances between these two groups: Actinobacteroita and Proteobacteria increased in tumor samples, while Firmicutes, and to a lesser extent Cyanobacteria, were more present in adjacent normal tissues. At the genus level, *Streptomyces*, *Cloacibacterium*, and *Corynebacterium* showed a higher prevalence (15). Differently, the genus *Acholeplasma* was the most predominant in the adjacent normal tissues, followed by *Streptomyces*. Further microbiome analysis based on the categorizing of the tumor group into three segments based on differentiation (G1 - well differentiated, G2- well to moderate/moderately differentiated, and G3/G4 - poorly differentiated) was carried out with the aim to explore the relationship between bacterial abundance and clinical features. The bacterial genera, *Delftia* and *Staphylococcus*, were the most abundant at the G1 stages compared with G2 and G3/G4 stages. On the other side, *Actinobacter* and *Cloacibacterium* were the most abundant genera in G2 and G3, respectively. The relative abundance of *Delftia* in G1 suggests its potential role in tumor development and progression paving the way for further exploration as a potential molecular signature for disease diagnosis.

Riquelme reported higher alpha-diversity in the PDAC microbiome of LTS patients and identified an intra-tumoral microbiome signature (*Pseudoxanthomonas/Streptomyces/Saccharopolyspo/Bacillus clausii*) highly predictive of significantly better outcomes in both cohorts selected from two different hospitals and used for discovery and validation (22). The LTS tumors exhibited an enrichment of Alphaproteobacteria, Sphingobacteria, and Flavobacteria at the class level. Differently, the STS tumor cases were dominated by Clostridia and Bacteroidea. The results at the class level agree with those obtained by Huang et al., mentioned above. PDAC specimens from LTS patients showed a predominance of Proteobacteria (Pseudoxanthomonas) and Actinobacteria (Saccharopolyspora and Streptomyces), while no predominant genus was detected in the STS tumors (**Table 2**). Based on these considerations, the authors speculate that the presence and abundance of the three taxa Saccharopolyspora (*Saccharopolyspora rectivirgula*), Pseudoxanthomonas and Streptomyces, together with the presence of *Bacillus clausii*, could influence and predict long-term survivorship in PDAC patients (22).

Zhang and co-authors evaluated the possible impact of intratumoral microbiome on the progression of PDAC by storage PDAC tissue along with matched peritumoral tissue samples, demonstrating that they share similarities, but also some differences (20). In detail they detected no differences in alpha diversity between the tumor and peritumoral tissues, whereas the beta diversity analysis showed that the microbial distribution in tumor tissues and paired peritumoral tissues was highly similar. At the phylum level, both tumor and peritumoral tissues microbiomes were represented by Proteobacteria and Firmicutes, although a significant difference was observed in their abundance: in peritumoral tissues they observed a predominance of Proteobacteria, expecially Gammaproteobacteria, whereas in PDAC tissues Firmicutes were markedly more prevalent. Remarkably, at the genus level, a higher abundance of *Exiguobacterium* and *Stenotrophomonas* in tumor tissue than in peritumoral tissue, was detected. Similar to Huang et. al. the authors divided the patients in two categories, based on the survival. LTS group exhibited a higher relative abundance of *Bacillus* (genus level) in both tissue samples compared to the STS group and a lower abundance of *Exiguobacterium* in the peritumoral tissues of the LTS group compared to the STS group. Tissue samples from STS patients showed a dominance of Deinococcus_Thermus (phylum level) and Deinococci (class level) (**Table 2**). Based on these results, the authors hypothesized a possible better outcomes for PDAC patients with a higher abundance of *Bacillus* and lower abundance of *Exiguobacterium* in tumor tissues (20).

Yu et al., integrated 582 samples derived from PDAC tissues across four datasets and presented a landscape of cancer microbiome at the genus level based on RNA-Seq data (39). On average, there were hundreds of genera distributed in the PDAC tissue, and dozens of core microbiome were identified by PDAC tissue. The pan-microbiome tissue of PDAC, estimated at more than 2,500 genera, was also evaluated. Firmicutes, Proteobacteria, Actinobacteria, and Bacteroidetes were the most prevalent and abundant phylum across the four datasets. 56, 54, 20, and 22 genera were considered core microbiome in each dataset. 54 core microbiomes were identified across the four datasets, among which the genera *Kocuria*, *Streptococcus*, *Bacillus*, *Lactobacillus*, *Ralstonia*, *Staphylococcus*, *Acinetobacter*, *Pseudomona*s were shared. Nevertheless, the relative abundances of each genus varied greatly among the 4 datasets. Among the top 20 taxa, Gammaretrovirus and Ralstonia were the most coefficient taxa with mice patient-derived xenografts (PDX) samples and human tissue samples, respectively. Moreover, 17 of the 20 taxa proved significant differences in relative abundance between two datasets, which correspond to a set of human tissue samples and mice PDX samples, respectively. The relative abundance of Gammaretrovirus in PDX samples was 376 times higher in average compared to that in human tissue samples. In PDX samples, *Staphylococcus, Ralstonia, Moraxella and Cloacibacterium* also showed higher abundance, whereas *Corynebacterium, Streptococcus, Bacillus, Pseudomonas, Acinetobacter, Salmonella, Massilia, Lactobacillus, Halomonas, Pantoea*, and *Clostridium* were highly abundant in human tumor tissue samples. Generally, human tissue samples had significantly higher values of microbial richness and divergence than PDX samples (39).

Since microbiota refers to all the microorganisms identified in the human body, including bacteria, archaea, viruses and fungi, we also analyzed articles related to fungi and virus reported in PDAC microenvironment.

PDAC tumors are infiltrated mostly by fungal genera such as *Alternaria* and *Malassezia* (40, 41). According to Alam and co-authors, intratumor fungi facilitate the secretion of IL-33 from PDAC cells and accelerate PDAC tumor growth. To further validate this statement, the authors demonstrated that genetic deficiency of IL-33 or an anti-fungal treatment reduced tumor burden and increased survival in a preclinical mouse model. This study poses new highlights into mechanisms driving PDAC tumor progression and into the identification of therapeutic pathways involving intratumoral mycobiome-driven secretion of IL-33 (41). Aykut et al. showed that PDAC mice and human samples presented a ~3000-fold increase in fungi compared to normal pancreas. The composition of the PDAC mycobiome was distinct from that of gut or normal pancreas based on alpha and beta diversity indices. The fungal community within PDAC tumors was enriched for *Malassezia* in both mice and human samples. Furthermore, the authors investigated the possible mechanism of action of fungi in promoting pancreatic tumor. They first observed that fungal ablation was tumor-protective in slowly progressive and invasive models of PDAC whereas repopulation with *Malassezia* – but not *Candida*, *Saccharomyces*, or *Aspergillus* – accelerated oncogenesis. In detail, fungal wall glycans bind mannose-binding lectin (MBL) activating the complement cascade which promote tumor progression. Moreover, MBL deletion in the extra-tumoral compartment in tumor cells were protective (40).

The association of virus species with pancreatic cancer is controversially discussed, especially for human herpesvirus 6 (HHV-6). A recent study analyzed 101 samples of patients with PDAC in tumor and in surrounding normal pancreatic tissue and unveiled that the 68% of all patients were HHV-6 DNA positive in any of the samples and 49% were positive in tumor tissue (38). All specimens except one were positive for HHV-6B but the authors hypothesized that HHV-6 DNA-positivity of pancreatic cancer tissue was probably not due to the infection of pancreatic cells by HHV-6, but to the migration of HHV-6 positive immune cells into the pancreas. The authors concluded there was no direct evidence that HHV-6 was the causative agent of pancreatic cancer, and that further studies were necessary to establish the connection. Another study published in the selected range of years showed that infection with the SARS-CoV family could increase the risk of the tumor development by altering the expression of different oncoproteins. The findings suggest the PDAC as the most possible malignancy occurring after sever infection with SARS-CoV family (42).

## 4 Is microbiota involved in early step of PDAC tumorigenesis?

Evidence from the literature indicates a distinct alteration in the microbial composition of pancreatic and peripancreatic tissues during the progression of PDAC, starting from early premalignant lesions such as Intraductal Papillary Mucinous Neoplasms (IPMNs) and Pancreatic Intraepithelial Neoplasia (PanIN).”

**Figure 2:**
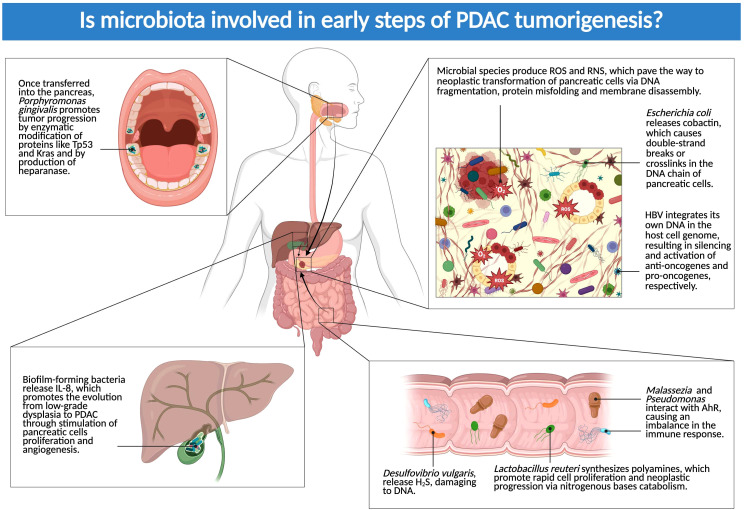
Is microbiota involved in early steps of PDAC tumorigenesis? The figure above showcases the main locations where microbial species can exert their influence on the early phases of PDAC development. Dysbiosis, both in the tumor and in distant sites (biliary tract, gut, and oral cavity), contributes to the progression from pretumoral lesions (IPMN and PanIN) to proper carcinomas (Created in BioRender.com).

Mechanisms of the crosstalk between pre-tumoral cells and the microbiota will be unveiled in the following sections (**Figure 2**).

### 4.1 Intratumoral and biliary tract microbiota and PDAC

#### 4.1.1 Intratumoral microbiota in pre-tumoral lesions

Several recent studies have highlighted an alteration of microbial populations in the cystic fluid of IPMNs. For instance, when compared with other pancreatic cystic neoplasms (PCNs), IPMNs with high-grade dysplasia showed an higher level of intracystic bacterial 16s DNA copy numbers. Indeed, an increased prevalence of bacterial strains from the oral cavity was reported in IPMN cystic fluid samples, including *Fusobacterium nucleatum* and *Granulicatella adiacens* (23).

Moreover, it was discovered that PMNs with both low-grade and high-grade dysplasia harbor gut-derived bacterial phyla like *Firmicutes* or *Proteobacteria* (24).

While it appears that microbes have a natural predisposition for pancreatic colonization, it should be noted that medical procedures also have a role in causing and accelerating colonization and dysbiosis: endoscopic retrograde cholangiopancreatography and biliary stenting may open a way for gut microbial flora to migrate into the biliary tree through induced reflux (24). These procedures are not exclusive to oncologic patients, but they are also recurring in non-oncologic patients suffering from PDAC - predisposing conditions, like pancreatitis and other biliary tract diseases. Therefore, one should keep in mind the potential pro-inflammatory and carcinogenic effects of these procedures when suggesting them to cancer-free patients with a higher risk of developing PDAC.

Based on these considerations, the current review will focus on the different microbial species in the tumoral and peritumoral environment as showed in recent literature.

Moreover, biliary tract bacteria can influence the development and progression of pancreatic cancer, particularly PDAC, through immune, inflammatory, and metabolic mechanisms. The bile ducts and pancreas are closely connected anatomically, which facilitates microbial migration between these compartments, especially in the presence of cholestasis, biliary stenting, gallstones, or periampullary tumors. More specifically, biliary bacteria can contribute to a chronic inflammatory state that promotes neoplastic transformation. Indeed, Toll-like receptors (TLRs) signal induced by pathogens, activates pathways such as NF-κB and STAT3, promoting cell proliferation and inhibition of apoptosis 24.

Two of the earliest effects of microbial replication and multiplication in the pancreas are inflammation and increased oxidative stress: microbial species are responsible for the production of reactive oxygen species and reactive nitrogen species, which pave the way to neoplastic transformation of pancreatic cells via DNA fragmentation, protein misfolding and membrane disassembly (24).

Oxidative stress has been proven to play a determining role in the pathogenesis of inflammatory pancreatic diseases, which are known pre-tumoral conditions. While ROS scavengers (e.g. glutathione, superoxide dismutase, catalase, vitamin A, vitamin C, vitamin E, thioredoxin) are constitutively expressed by healthy pancreatic cells, their levels seems to be altered in patients with acute and chronic pancreatitis (43). Furthermore, the said diseases appear to be characterized by the impaired enzymatic activity of the molecules which are responsible for the generation of ROS/RNS. These molecules include cytoplasmic Xanthine Oxidase and Nitric oxide synthase, which is generally expressed in the plasmatic membrane of PC but may also be localized in the nucleus, the mitochondria or the Golgi apparatus (43). Monooxygenases of the family of Cytochrome P450, typically located in the smooth endoplasmic reticulum, also seem to the involved in pancreatic oxidative inflammation, as it is demonstrated by their higher prevalence in pre-cancerous ethanol-induced chronic pancreatitis (43).

Some of the pancreatitis-determining oxidative enzymes were stated to be active in the pathogenesis of PDAC, indeed. NADPH oxidase is massively expressed in the plasma membrane of pro-inflammatory neutrophils and macrophages of PDAC patients (43, 44). Since dysbiosis promotes the neutrophils and macrophages - mediated immune response, it is reasonable to hypothesize its involvement in the genesis of oxidative stress also via immunomodulation.

Bacterial and viral species, such as *Escherichia coli* (24) and *Hepatitis B Virus* (HBV) (25) may play a direct role in altering the genetic structure of pancreatic cells. After being internalized in the host pancreatic cell, *E. coli* releases the bacterial toxin colibactin in the cellular cytoplasm, which causes double-strand breaks or crosslinks in the DNA chain. These alterations in the genome structure are a major cause of intracellular inflammation, which is further aggravated by the endocytic release of digestive enzymes by the damaged cells (24). HBV integrates its own DNA in the host cell genome as part of its reproductive cycle; the integration results in silencing, both selective and random, of anti-oncogenes and activation of pro-oncogenes, respectively. While HBV may remain in a quiescent state, intracellular expression of its procarcinogenic viral proteins (e.g. regulatory protein X) has been observed at later stages of PDAC (25).

#### 4.1.2 Biliary tract bacteria effects

Arteta et al. have recently developed a potential reliable model of bacteria-related carcinogenesis in the biliary tract, which may be applied to all hepatobiliary tumors, including PDAC. Promoted by obesity, diabetes mellitus, smoke and genetic predisposition, inflammation arises in the gastrointestinal (GI) tract, leading to local dysbiosis. Once microbial dysbiosis is established, it is further protracted by increased levels of antimicrobials, which result in a reduced diversity in bacterial species in favor of pro-tumorigenic ones. The latter tend to form biofilm in the biliary tract, as proven by the increased expression of quorum sensing-associated bacterial proteins (e.g. zeatin and surfactin). Biofilm-forming bacteria produce and release IL-8 in the extracellular environment which is not only capable of extending dysbiosis, but also of promoting the evolution from low-grade dysplasia to PDAC through stimulation of pancreatic cells proliferation and angiogenesis (45).

Moreover, several studies highlighted the frequent presence of specific bacterial species in the bile ducts of patients with pancreatic cancer or underlying predisposing conditions. One of the most commonly identified is *Enterococcus faecalis*, a bacterium frequently involved in biliary infections. It is known to produce free radicals, which may lead to DNA damage, to the development of chronic pancreatitis and, subsequently, pancreatic cancer (46).

*E. coli*, a Gram-negative bacterium, is also often found in such cases and it is well known for its role in cholangitis. It produces lipopolysaccharides (LPS), which activate the Toll-like receptor 4 (TLR4) and trigger chronic inflammation, thereby contributing to a tumor-promoting microenvironment (47).

*Klebsiella pneumoniae* is another frequently detected pathogen in biliary infections, particularly in patients with biliary stents or prostheses. It may promote local inflammation and immune suppression, factors that can facilitate tumor progression.

*Fusobacterium nucleatum*, previously associated with gastrointestinal tumors, has also been implicated in pancreatic cancer. It can stimulate tumor cell proliferation and migration via autocrine and paracrine signaling mechanisms, while also inhibiting the local immune response (48).

Lastly, bacteria from the *Helicobacter* genus—specifically *Helicobacter bilis* and *Helicobacter hepaticus*—are considered potentially carcinogenic. These microorganisms are capable of inducing chronic inflammation in the biliary tract, possibly contributing to both hepatobiliary and pancreatic carcinogenesis.

### 4.2 GI tract microbiota and PDAC

#### 4.2.1 Gut microbiota

The pancreas is not an isolated system: microbes from adjacent organs, mainly the proximal GI tract, are involved in a biochemical cross-talk with the biliary environment. Further knowledge is required in order to establish a clear cause-effect relationship between GI microbes and PDAC, but the few results gathered so far have been promising.

Mendez et al. performed a stool analysis in mice who had not developed observable PDAC yet, but had PanIn with various level of dysplasia: the team observed microbial dysbiosis, with a higher relative abundance of *Clostridia*, *Bacilli* and *Erysipelotrichia*, *Actinobacteria*, and *Deferribacteres.* This could prove a positive association, with a chronological order, between higher dysbiosis rates and the development of premalignant pancreatic neoplasms (33).

With regard to pre-tumoral chronic pancreatitis, the change in gut and pancreatic microbiome plays a significant role in its neoplastic progression. Microbial dysbiosis can influence various aspects of pancreatic carcinogenesis, especially immunomodulation and inflamation. Chronic pancreatitis is characterized by persistent inflammation of the pancreas. Intestinal dysbiosis can exacerbate this inflammation. In particular, an increase in pathogenic bacteria such as Escherichia-Shigella and Klebsiella, and a release of beneficial bacteria such as Bifidobacterium have been observed in patients with CP and PDAC compared to healthy controls (49).

#### 4.2.2 Oral microbiota

Other suspects of microbial-induced carcinogenesis were also identified in the oral cavity, where *Aggregatibacter actinomycetemcomitans* and *Porphyromonas gingivalis* are the most studied species, along with *Bacteroides* spp., and *Granulicatella adiacens.*

Although no clear clue is available on how oral bacterial species may migrate through the GI tract and into the pancreas, there is a sequential correlation between *P. gingivalis* infection and tumor induction via enzymatic (peptidyl-arginine deaminase) modification of proteins like TP53 and KRAS. Patients also showed elevated antibodies against *P. gingivalis* years before their PDAC diagnosis (34). Of note, the hypoxic PDAC microenvironment is a vantage point for *P. gingivalis*, as it proliferates when cultured in human Pancreatic cancer (PC) cells incubated in 1% oxygen. It also stimulates production of heparanase, a potential protumoral enzyme that stimulates the reproduction of cancer cells (50).

#### 4.2.3 Microbial metabolites

An imbalanced diet, rich in fat and meat-derived proteins, is a universally recognized risk factor for PDAC development. Combined together, distal colon dysbiosis and an unbalanced diet can provide a favorable environment and substrates for cancer development: an excess in dietary proteins and animal fats causes a shift from a saccharolytic to a more proteolytic fermentation, with an accumulation of pro-neoplastic nitrogen compounds, such as phenols and ammonia. On top of that, some proliferating bacteria, such as *Desulfovibrio vulgaris*, release hydrogen sulphide, causing DNA damage (51).

Indeed, a connection between gut dysbiosis and metabolism pathways alteration in pre-tumoral cells was recently discovered: 4-month-old mice showed an increased concentration of polyamines (Putrescine, Spermidine and Spermine), metabolites responsible for rapid cell proliferation and neoplastic progression via purine and pyrimidine nitrogenous bases catabolism. As tumors progress from PanINs to PDAC, an increase abundance of *L. reuteri*, bacterium associated with polyamines metabolism, has been detected. Even though *Lactobacillus* spp. are able to influence polyamine metabolism and growth inhibition in gastric cancer, their role in PDAC remains unknown (33).

The GI fungus *Malassezia* has been proved to often translocate to the lipophilic pancreas, probably since it lacks the enzymes responsible for fatty acid synthesis and relies on external sources for survival. It also produces indoles, with a broad effect on our immune system: these compounds interact with the aryl hydrocarbon receptor (AhR), causing an imbalance in the immune response, and aggravating pro-tumoral characteristics of the TME. A similar effect on AhR has also been discovered analyzing human PDAC samples colonized by gut-derived *Pseudomonas* genus, in this case via phenazine pigments (28).

## 5 How does the microbiota boost PDAC progression?

**Figure 3:**
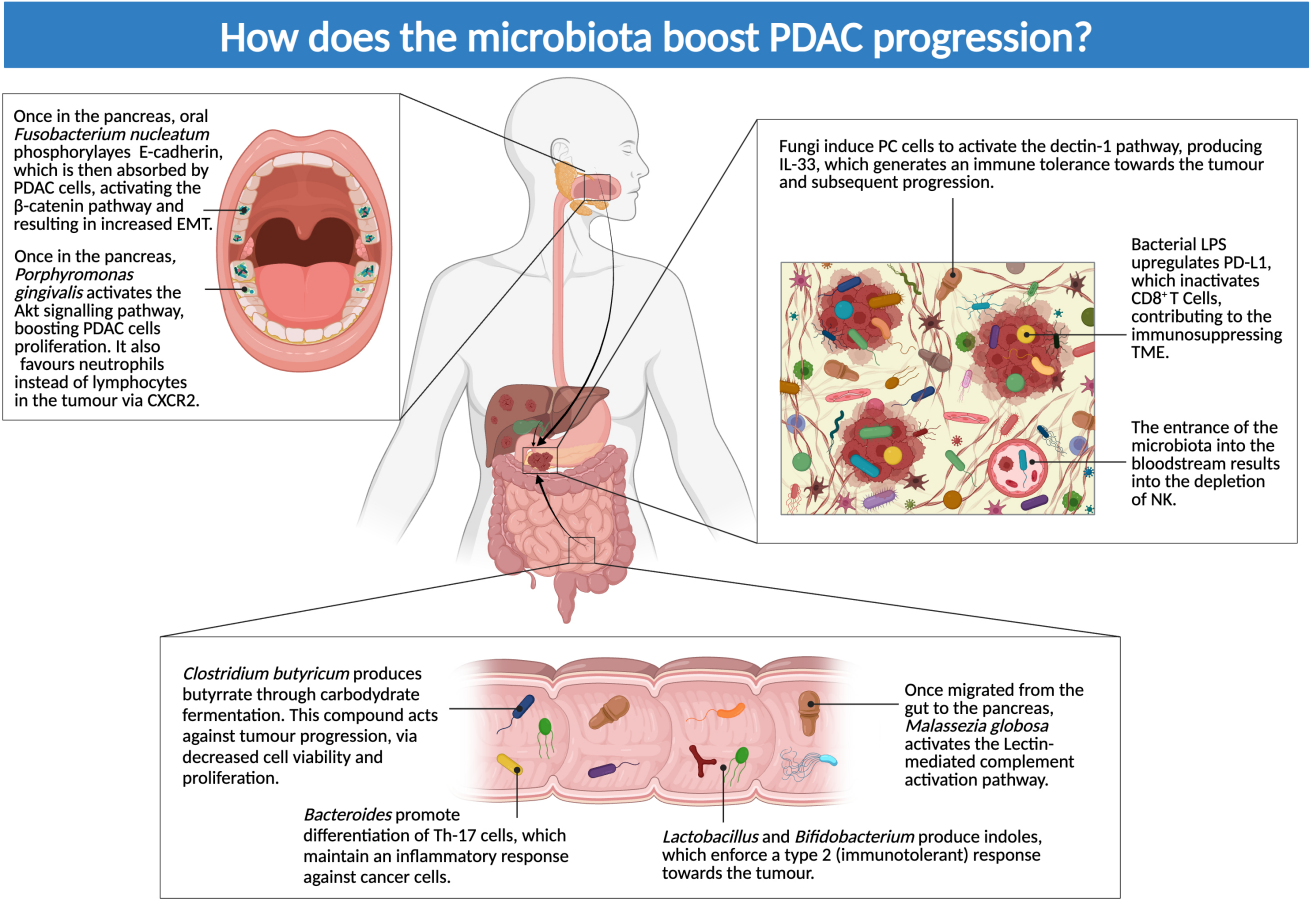
How does microbiota boost PDAC progression? The figure above illustrates dysbiosis-mediated mechanisms of local and distant disease progression: microbes seem to have both a direct effect on PC cells and an influence on the immune system, driving it toward an immunotolerant phenotype (Created in BioRender.com).

The impact of microbiota alterations is not only limited to tumorigenesis, but dysbiosis may also be involved in both local and distant disease progression (**Figure 3**). Moreover, microbiota can influence PC directly in both favorable and harmful ways.

### 5.1 Direct and indirect effects of crosstalk between microbes and cancer cells

*F. nucleatum* has been shown to infect pancreatic tissue, causing direct and indirect consequences on the advance of PDAC. *F. nucleatum* phosphorylates E-cadherin, which is then absorbed by cancer cells: the phosphorylation activates the β-catenin pathway, resulting in increased epithelial mesenchymal transition (EMT) (26). Granulocyte-macrophage colony-stimulating factor (GM-CSF), IL-8 and Chemokine (C-C motif) ligand 20 (CCL20) or liver activation regulated chemokine (LARC) or macrophage inflammatory protein-3 (MIP3A) are also produced by the infected cells, generating an inflammatory environment (29).

Likewise, *P. gingivalis* is directly involved in the proliferation of PDAC via activation and augmentation of the Akt signaling pathway (**Figure 3**). *P. gingivalis* benefits from cell survival, given that it is an intracellular microorganism (51). Alam et al. reported a difference between antimycobial-treated and untreated mice with PDAC in regards to a dectin-1 pathway, mediated by PC cells: this molecule is implied in NF-kβ activation pathway. PC cells exposed to fungal components activate this pathway and secrete IL-33, generating an immune tolerance toward the tumor and subsequent progression. This was proven by the fact that treatment with Amphotericin B led to a decrease in this pathway, therefore establishing the cause-effect relationship between fungi and cancer cells survival (41).

Metabolites are an indirect way for microbes to influence their surroundings: these molecules may be picked up by nearby or distant cells, with both protumoral and antitumoral effects. Butyrate is derived from carbohydrate fermentation performed by bacterial cells in the gut, mainly *Clostridium butyricum*: this compound may act against tumor progression. In a study Yang et al, external administration of butyrate led to a decrease both in cell viability and proliferation. These results were accomplished both *in vitro* and *in vivo* (27).

On the other hand, *Pseudomonas* likely exerts a pro-tumorigenic effect through the synthesis of phenazine pigments. These byproducts act as an indirect immunosuppressive factor when they come in contact with AhR in macrophages: this interaction activates an immunosuppressant pathway, which favors cancer progression (28).

### 5.2 Immunomodulation

Manipulating the immune system is widely adopted as a survival strategy by PDAC-associated microbes. However, the imbalance generated in the immune response leads indirectly to prolonged cancer survival and expansion, with an overall worse prognosis for patients.

*Malassezia globosa* is a prime example of this: the fungus is capable of migrating from the gut to the pancreas, as it was observed in mouse models (28). *M. globosa* positive samples from PDAC patients, premalignant lesions, or subjects with chronic pancreatitis, showed an elevated inflammatory response, mainly the lectin-mediated complement activation pathway (30). Even when the abundance of *M. globosa* did not differ between healthy and sick samples, it has been reported that specific cancer-related genes had a higher activation rate in sick patients with *M. globosa* positivity (52). Absence (in knockdown mice) or suppression (via antifungal treatment) of this pathway seems to prevent the advance of PDAC and other tumor types (40, 41). Hezaveh et al. confirmed that indoles produced from certain *Lactobacillus* or *Bifidobacterium* strains acted as an immunosuppressant, enforcing a type 2 (immunotolerant) response toward the tumor; this led to an overall worse prognosis in patients enriched with these strains (31). This relationship was confirmed when ablation of AhR increased macrophage and T Cell response (29).

A similar effect was reported by observing toll-like receptors (TLRs), activated by interaction between cancer cells and the TME microbiota: in particular, TLR 2 and 5 alter macrophage activity, favoring an immunosuppressive phenotype, while also inhibiting cytotoxic T-Cells; TLR 4 binds LPS, activating dendritic cells and stimulating tumorigenesis; lastly, TLR 9 attracts regulatory T cells and Myeloid-derived suppressor cells (MDSCs) and stimulates activation of pancreatic stellate cells (PSCs) (32).

Lipopolysaccharide (LPS) seems also involved in preventing infiltration of immune cells in PDAC tissue, via upregulation of PD-L1 which inactivates CD8^+^ T Cells (24). A similar effect is carried out by *P. gingivalis*, which favors neutrophils instead of lymphocytes in the tumor via (CXC motif chemokine receptor 2 (CXCR2) (29) (**Figure 3**).

Another aspect that needs further investigation is handling tumor expansion and metastasis: this function is normally performed by natural killer cells. This population seems decreased when microbiota causes a bloodstream inflammation; this scenario can also happen in patients with cachexia or other chronic inflammations, with an impairment in the adaptive immune response (24).

However, one must not consider the microbiota as an intrinsically negative entity: *Bacteroides* species might be involved in the correct differentiation of immune Th-17 cells, which maintain an inflammatory response against cancer cells. A deprivation of *Bacteroides* led to a reduction in the Th-17 population, impairing the inflammatory response (53) (**Figure 3**).

## 6 Current application and possible future uses of microbiota

**Figure 4:**
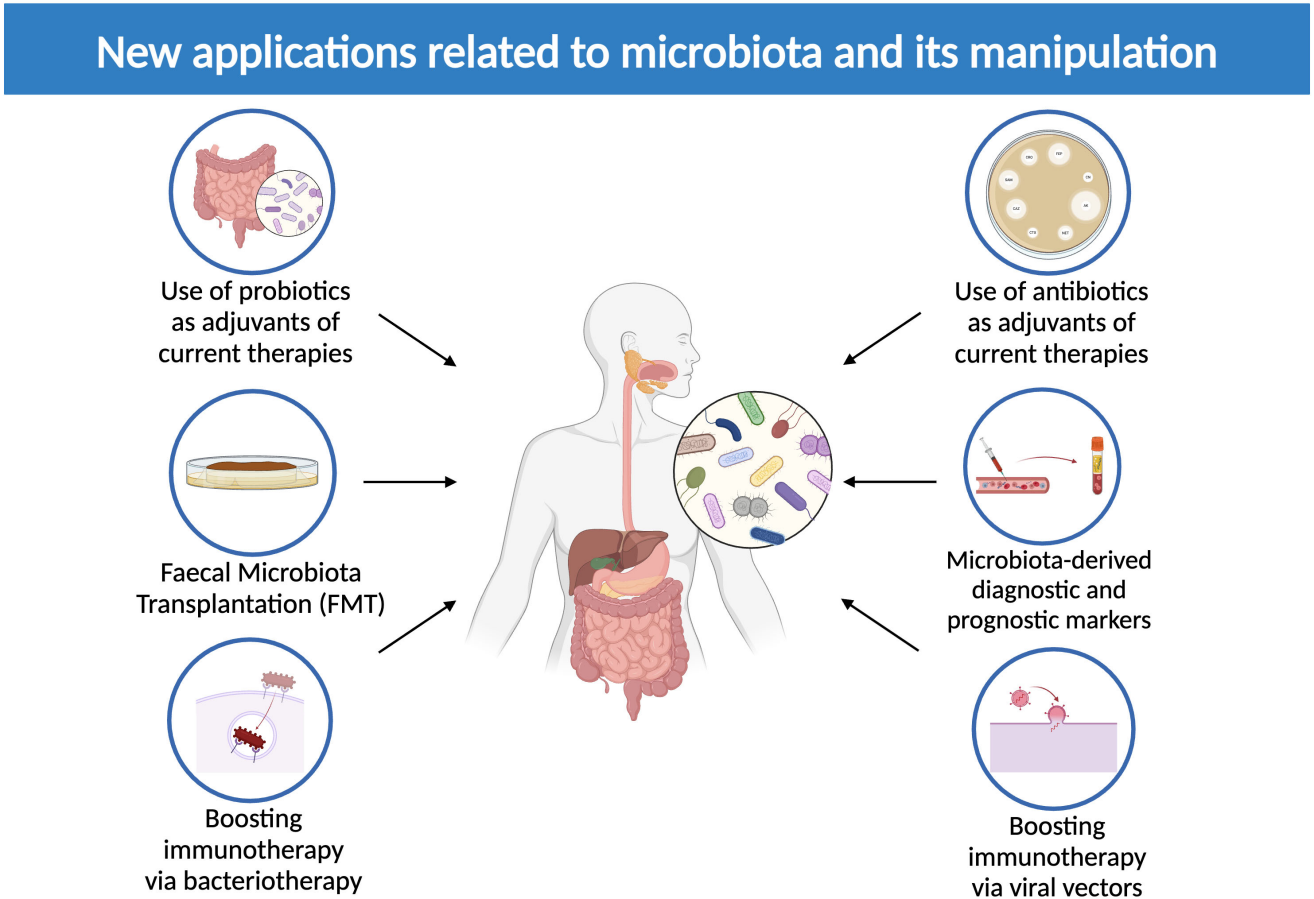
New applications related to microbiota and its manipulation. The image above displays currently available microbiota-related therapeutic options, including the use of probiotics and antibiotics to adjuvate current therapies, microbiota-derived diagnostic and prognostic markers, fecal microbiota transplantation (FMT) and the employment of bacterial and viral vectors to boost immunotherapy (Created in BioRender.com).

Progressively increased interest has been shown toward a possible conversion of intratumoral and extratumoral microbiota into a tool against cancer: microbes are suitable means of early diagnosis, prognosis assessment and therapeutic implications (**Figure 4**).

Microbiota modulation through probiotics and antibiotics has emerged as a way to modulate tumor growth and support therapeutic protocols (51, 54–56). More specifically, the prospect of employing specific probiotics as a way of preventing tumor progression and metastasis has been recently highlighted (51, 54–56).

Antitumoral-butyrate-releasing bacteria *Faecalibacterium prausnitzii*, *Eubacterium rectale* and *Roseburia intestinalis* are scarcely represented in early-stage PDAC samples (54), therefore they constitute consistent options as components in a probiotics mixture to be administered to early-stage PDAC patients.

Indeed, when administered with probiotics *Bifidobacterium* spp. and *Lactobacterium* spp. *per os* in combination with chemotherapy, PDAC-bearing mice showed reduced metastatic potential due to delayed EMT (54). Moreover, supplementing probiotics *Bacteroidales* and *Burkholderiales* to PDAC xenograft mice, previously treated with Ipilimumab, ameliorated collateral colitis (51).

The function of adjuvant chemotherapy, in terms of both amplifying its efficacy and preventing its aftereffect reactions, may be obtained also through antibiotics.

The evidence that Gammaproteobacteria species, including *Enterobacter* and *Pseudomonas*, are capable of inactivating gemcitabine in 2′,2′-difluorodeoxyuridine with cytidine deaminase, inspired Nakano et al. to administer antibiotics to late-stage PDAC patients alongside with Gemcitabine and Nab-paclitaxel. This resulted in better prognosis, as proven by the increased progression-free survival (PFS) and overall survival (OS) rates (56). Analogously, receiving antibiotics in association with Gemcitabine or 5-fluorouracil raised OS and cancer-specific survival (CSS) rates of a large cohort of metastatic PDAC patients as evidenced in a recent retrospective cohort study (57).

Furthermore, antibiotics have been proved useful in alleviating diarrhea caused by Irinotecan hydrochloride, a constituent of mFOLFIRINOX, the current first-line treatment for PDAC. In the liver, carboxylesterases transform Irinotecan hydrochloride into SN-38, an active metabolite which is toxic to the epithelial cells that constitute the internal lining of the intestinal wall. Since gut dysbiotic flora prolongs Irinotecan toxicity by re-transforming inactive metabolite SN-38 G into its active form SN-38, it is reasonable to think that preventing gut dysbiosis through antibiotics may be beneficial for PDAC patients. Since bacterial classes Enterobacteriaceae and Clostridium are responsible for Irinotecan-related diarrhea, PDAC patients could be treated with antibiotic therapy (51).

### 6.1 The microbiota as a new set of tumor markers

While the microbiota-PDAC relationship is being adequately examined and defined, it would seem reasonable to explore the possibility of sourcing useful microbial molecules, in the prospect of employing them as predicting factors of both early-stage PDAC development and late-stage PDAC progression and metastasis. Indeed, recent literature provided us with compelling works regarding microbiota-derived diagnostic and prognostic markers, which will be discussed in the following paragraphs.

#### 6.1.1 Microbiota-derived diagnostic markers in PDAC patients

Two up-to-date studies have examined the connection between GI-tract microbial species and early-stage PDAC, suggesting the opportunity of recruiting digestive system flora as a means of early diagnosis.

In particular, when analyzing the saliva and the stool of Spanish and German patients with PDAC, Kartal et al. detected 27 fecal bacterial species that are suitable for cancer detection both during its most precocious stages and in a more advanced state. Associating the presence of these specific bacteria with well-known marker CA19-9, further elevated their accuracy (58). Additionally, Nagata et al. identified 30 gut and 18 oral microbial species, including bacteriophages, which were correlated with early-stage PDAC in Japanese patients (59). Although further investigation on the current matter is required, the fact that they were performed on two heterogeneous cohorts of patients corroborates the reliability of these studies.

The oral cavity seems to be the optimum environment to probe in order to find possible microbial candidates that could suggest PC development. The tongues of patients suffering from pancreatic head cancer displayed a different bacterial composition, not only in comparison with healthy subjects, but also with patients with liver cancer. This suggests that some bacterial species may be used both to detect PDAC precociously (*Haemophilus*, *Porphyromonas*, *Leptotrichia* and *Fusobacterium* spp.) and to distinguish it from liver cancer (*Streptococcus* spp.) (60). A particular interest by the scientific community was expressed toward *P. gingivalis* and *A. actinomycetemcomitans*, the main bacterial species that cause periodontal diseases. A strong correlation between periodontopathies and PDAC was highlighted, to the point that patients with periodontal diseases have a 64% higher risk of developing PDAC, especially non smokers (23). Additionally, it was possible to find *P. gingivalis* and *Aggregatibacter* spp. antibodies in the plasma of patients with PDAC, highlighting a new prospect of early PC diagnosis via liquid biopsy (50).

Proceeding from the proximal to the distal end of the GI tract, gut microbiota is another precious source of diagnostic markers, with particular regard to bacterial and fungal populations. As previously discussed, PDAC patients show a depleted intestinal flora in terms of bacterial species variety, with the prevalence of pathogens like *Bacteroides* (61) and *Fusobacterium* spp (62). On the contrary, helpful bacteria, such as *Firmicutes* and *Proteobacteria* (61) and *Lactobacillus* spp (62), seem to be less abundant than in healthy subjects. Furthermore, the microbial composition of the duodenal fluid in patients with PC was significantly altered in comparison with healthy controls and subjects with benign pancreatic cysts, with increased levels of *Bifidobacterium* genera (63). Therefore, a way of hastening PC diagnosis could reasonably be to research these pieces of evidence in PDAC patients’ gut and duodenal fluid microbiota. An additional plausible way of microbiota-derived early PC diagnosis is via microbial metabolites. In this regard, recently Zhong et al. identified five possible novel markers of PDAC, of which X-21849 showed inverse proportionality with the levels of *Flavonifractor* sp*90199495*, suggesting that the levels of the above-mentioned flavonoid-degrading bacterium are positively associated with PDAC (64). Also, Jakob et al. discovered two valid markers of both early-stage and late-stage PDAC: KIF5B and SFRP2, which not only seem to be hyperexpressed in PanINs and PDAC, but also in distant metastases. In particular, SFRP2 levels are controlled by gut flora, including *Bifidobacterium* spp., that are proven to increase in levels during the development of PDAC (65).

#### 6.1.2 Microbiota-derived prognostic markers in PDAC patients

PDAC TME harbors a plethora of microorganisms, to which the potential of predicting PDAD natural history and clinical outcomes was lately attributed. A pivotal article by Riquelme et al. suggests that pancreata of long-term survivors are enriched with bacterial classes Alpha-proteobacteria, Sphingobacteria, Flavobacteria, and bacterial genera of *Proteobacteria* (e.g. *Pseudoxanthomonas*) and *Actinobacteria* (*Saccharopolyspora* and *Streptomyces*). They presumably contribute to ameliorate PDAC natural history by boosting the molecular pathway that lead to degradation of xenobiotics, other than amino acids, lipids, terpenoids and polyketides. Short-term survivors, instead, bear higher levels of Clostridia and Bacteroidea in their pancreata (22). Additionally, positive clinical results in PDAC patients seem to be more frequent in case of higher levels of *Bacillus* inside the tumor and lower concentration of *Exiguobacterium* in the peritumoral tissue, with lower *Exiguobacterium*/*Bacillus* ratio (20).

By contrast, tumor-derived *Fusobacterium* spp (66). and bile-derived *Klebsiella pneumoniae* (67) were connected to poor prognosis, in terms of CSS and PFS respectively. Other than intratumoral microbiota, gut flora seem to be a reliable source of prognostic markers in patients with PDAC. Of note, collecting gut microbiota is definitely much more feasible and safer than collecting intratumoral samples, given the fact that it does not require a fine needle aspiration (FNA) biopsy of the pancreas nor a surgical excision. Considering the technical complexity of these procedures and their possible side effects, the advantages of exploring gut microbiota instead of the intratumoral one would seem clear.

Several GI bacterial species were correlated with good and bad prognosis in subjects with PC, which will be addressed as follows. The bacterial taxa Saccharopolyspora, Pseudoxanthomonas and Streptomyces and the species *Bacillus clausii* seem to be related to better prognosis, as they would seem to be more abundant in long-term survivors than in short-term ones. This could be explained by the fact that their presence results in higher concentration of CD8+ T cells in the plasma of patients with PDAC (22). On the contrary, taxa Bacteroides, Lactobacillus, and Peptoniphilus are associated with worse prognosis, probably since their levels are inversely correlated with the concentration of CD4^+^, CD8^+^, CD45RO^+^ T cells in PDAC (68). Also, 10 bacterial taxa (Kurthia, Gulbenkiania, Acetobacterium, Planctomyces, Xenophilus, Gardnerella, Advenella, Catenuloplanes, Leptolyngbya, and Proteus) could predict the tendency of recurrence and metastasis in PDAC patients, as confirmed by a decreased relative abundance of abovementioned bacteria in patients with metastatic PC. On the other hand, *Acetobacterium*, *Catenuloplanes*, and *Leptolyngbya* were enriched in those same patients (69). A bacterial genus of particular interest in this field is *Fusobacterium*, which was observed both in pancreatic tissue (66) and in duodenal fluid (63) of PDAC patients with poor prognosis.

Not only microbial species themselves may contribute to assessing PC clinical outcomes, but by-products of their metabolite have also shown potential usefulness in this matter. The gut metabolite butyrate is associated with better disease control, as its lower levels in fecal specimens result in increased tumor progression and chemo-resistance (29). Consistently with that, patients who received sodium butyrate *per os* in association with Gemcitabine had better outcomes in terms of tumor growth. Similarly, indole-3-acetic acid (3-IAA), a tryptophan metabolite in the gut, correlates with good prognosis, as regards of both PFS and OS. It contributes to PDAC chemo-response when oxidized by immune cells into toxic molecules (e.g. 3-methylene-2-oxindole), which amplify oxidative stress and halt autophagy in PDAC cells (29).

### 6.2 Advances in FMT

Several microbiota-based strategies are being clinically studied for other cancer types and could provide future directions for PDAC therapy. The most studied strategy is fecal microbiota transplantation (FMT), where fecal material from healthy donors is transferred to patients via endoscopy, colonoscopy or oral capsules. Numerous phase I trials demonstrated that FMT can induce favorable changes in the tumor microenvironment. One example is that obtained by Baruch and co-authors who detected a positive response in immunotherapy-refractory melanoma patients (70). In detail, the authors demonstrated that after FMT treatment, the microbiota compositions had a higher relative abundance of the immunotherapy-favorable Veillonellaceae family and a lower relative abundance of *Bifidobacterium bifidum*, reported to promote immune tolerance via T-regulatory cells. Moreover, the responders to immunotherapy, presented a higher relative abundance of Enterococcaceae, *Enterococcus*, and *Streptococcus australis*, and a lower relative abundance of *Veillonella atypica*.

Evidence supporting FMT approach in pancreatic cancer has been provided by Riquelme and co-authors, who showed that fecal material from LTS patients administered to murine PDAC models can be detected in the PDAC microbiota post transplantation and elicited anti-tumor immune activation (22). Transplanted microbiota can translocate into PDAC tumor and alter the TME, significantly reducing tumor growth. To further validate their hypotheses, the authors divided the transplanted mice into two groups and administered antimicrobials to one group. Interestingly the positive effect of FMT was lost if antimicrobials were administered, validating the central role of bacteria in reducing the tumor growth. Regarding the bacterial species, the authors detected differential clustering for beta-diversity between the two groups (22).

A phase I trial is ongoing to evaluate whether these findings can be translated into the clinical setting, with resectable PDAC patients receiving FMT delivered through colonoscopy or oral capsules [NCT04975217].

### 6.3 Microbiota-related therapies and immunotherapy

#### 6.3.1 Boosting immunotherapy via bacteriotherapy

Given the difficulty in finding effective therapies against PDAC, innovative strategies are being studied: these include techniques such as bacteriotherapy and viral vectors, in the effort of developing novel treatment protocols.

Wei et al. suggested that bacteria, such as *Bifidobacterium*, might have a beneficial role in strengthening GI wall integrity and immune response in patients with PDAC (71). Ebelt et al. reported a study in which an attenuated *Salmonella typhimurium* was equipped with hyaluronidase molecules on the cellular surface: given its affinity for the biliary tract, this bacterium would colonize the tumoral tissue and its surroundings, releasing hyaluronidase and dissolving the extracellular matrix surrounding cancer. Thus, the attenuated *S. typhimurium* expressing functional bacterial hyaluronidase would aid chemoimmunotherapy by granting an easier access to the target and fewer systemic side effects (72).

Intracellular *S. typhimurium* could also act as an immune system booster by delivering selected antigens to cancer cells, instead of drugs: when phagocytized by tumoral cells, *S. typhimurium* releases ovalbumin, which is then exposed on the cellular surface as an antigen. Ovalbumin renders cancer cells immunogenic and, therefore, they can be targeted by specific cytotoxic T cells aimed against ovalbumin. Thus, a specific immune response can be used to fight the tumor, in a drugs-sparing manner (73).

Another potentially useful bacterium is the heat-inactivated Group A *Streptococcus pyogenes*, heat-inactivated, given its capability to selective recognize and attach oncofetal fibronectin via streptococcal collagen like protein 1 (Scl1). A less risky way could be represented by using *Lactobacillus* specimens, engineered to express Scl1 and DNase Sda1, in order to obtain neutrophil extracellular trap dissolving bacteria without running the risk of a collateral infection (74). Moreover, Zhang et al., used a similar method to the previously cited teams: they modified *E. coli* to carry a combination of Doxorubicin (DOX) and Hydroxychloroquine (HCQ), and armed it with hyaluronidase on its surface. This allowed a penetration of the bacterium in the tumoral tissue; the release of DOX and HCQ combined the effects of chemotherapy (DOX mediated damage) and immune response (HCQ inhibits autophagy, resulting in an augmented antigen presentation) (75).

Bacterial metabolites may be a cheaper and, possibly, more accessible source of benefits against PDAC, since they are not live material and do not require culturing; this may have less unpredictable effects when tested on future patients.

Butyrate was previously discussed for its observed beneficial effects on tumors (28): two more short chain fatty acids, indole-3-acetic acid (3-IAA) and indole-3-propionic acid (3-IPA) may share these beneficial traits. In fact, 3-IAA acted as a chemotherapy boosting factor, while also showing antitumoral and anti inflammatory (relieving chemo-induced mucositis) effects *in vitro* (29).

Trimethylamine N-oxide (TMAO) is produced by CutC-synthesising bacteria (*Bacillus* and *Paenibacillus*), which have been associated with prolonged survival in PDAC patients. When put under observation by Mirji et al., TMAO demonstrated macrophage stimulation, polarizing tumor associated macrophages (TAMs) toward a type 1 antitumoral phenotype. The compound was also shown to inhibit immunosuppressive pathways frequently associated with the pancreatic TME. Upon coadministration with Immune Checkpoint Blockade (ICB) immunotherapy, the team observed augmented effects of ICB (76). While TMAO stimulated the innate response, a different metabolite could be used to boost the adaptive response: urolithin A (Uro A), obtained from pomegranate extracts modified by the gut flora, was capable of inducing a higher activation rate of T cells (both helper and cytotoxic) when added to an ICB protocol. This could lead to a stronger immunity toward cancer and metastasis, since Uro A induced formation of memory T cells against cancer cells (77).

#### 6.3.2 Boosting immunotherapy via antibiotic therapy

Unfortunately, not all bacteria are good bacteria: species like *F. nucleatum* and *P. gingivalis* are associated with earlier PDAC development and shorter OS. Keeping this in mind, an opposite approach, based on antibiotic therapy against harmful species, could be beneficial for patients.

Several studies support this hypothesis, reporting that ICB paired with antibiotics led to an increased tumor suppression: a possible explanation is that antibacterial and antifungal treatment removed harmful species (e.g. *Klebsiella* can inactivate Gemcitabine before it reaches the tumoral tissue), which would interfere with chemo and immunotherapy. Subsequently, removing this obstacle resulted in a boosted activation of T cells, a deeper tumor immune infiltration, and a better overall response to the applied protocols (23, 29, 34, 53, 71).

#### 6.3.3 Boosting therapy with viral vectors

Viral vectors have been a hot topic in scientific research for well over a couple of decades, with a rich history of theoretical applications: among these, some teams have explored strategies against pancreatic cancer.

Zhang et al., used a modified *Herpes simplex* strain (oHSV) which, when paired with anti CTLA and anti OX40 monoclonal antibodies (MAbs), managed to activate the host immune response against cancer cells, in a proinflammatory type 1 immune response (augmented antitumoral TAMs and effector T cells, and decreased regulatory T cells). However, ICB did not prove to be as effective as the combination of oHSV and Monoclonal Antibodies (78).

Another altered HSV strain, Myb34.5, proved effective when added to Gemcitabine. An unmodified strain, HF10, was tested on male Japanese PDAC patients with a 37% efficacy in tumor reduction and no reported side effects, with a positive association to immune activation in patients who received the vector, as demonstrated by increased levels of macrophages, CD4^+^ and CD8^+^ cells detected during autopsies (79).

Another phase I trial was performed on a combination of *Reovirus*, chemotherapy, and Pembrolizumab, bearing similar immuno-stimolatory effects (increase levels of intratumoral CD8^+^ T cells and of plasmatic CXCL9, CXCL10 and CXCL11) (80).

## 7 Conclusion

PDAC is, as of today, a complex disease to tackle: its subtle clinical evolution, lack of specific diagnostic tools and high aggressiveness make it a highly lethal type of cancer.

The tumoral microenvironment of PDAC proved to be just as complicated as a study subject, given the high variety of its cellular and acellular components: among these, the microbiota, both internal and external to the tumor, has been drawing attention in recent years. This review aimed at gathering more information about the possible roles and uses of the microbiota for clinical uses.

The microbial species inside the pancreatic tissue show a high variability in relation to various factors: patient age, diet, cancer stage and the treatments that patients undergo can all affect the composition in the microbiota. Although this review analyzed works on mice along with human models, evidence of microbial variability was consistent in both species.

Bacterial species were the most represented category of microbes in the consulted bibliography: these species were further subdivided by their site of origin, among intratumoral, GI tract, and oral cavity.

Regardless of origin site, all subdivisions of bacteria, and by extension, all microbial species inhabiting animal and human organisms, demonstrated a recurring pattern: a series of initial insults (unbalanced diet, alcohol, obesity, oxidative stress) makes up the base for dysbiosis, where potentially harmful strains (e.g. *Fusobacterium*, *Porphyromonas*, *Pseudomonas*) gain an advantage over otherwise predominant species in healthy species (listed in section 3). Dysbiosis, in turn, leads to harmful mechanisms perpetrated by microbial cells, through a cross-talk network between microbes and the surrounding environment. This can happen directly, via activation of pro-tumorigenic pathways (more specifically, Akt and β-catenin), or indirectly, with the production of bacterial metabolites (such as phenazine pigments, a byproduct of Pseudomonas with carcinogenic effects).

The data extracted from existing studies is likely still insufficient for drawing exact results, given the issues reported above (small cohorts and experiments carried out on mice), however, it is possible to conclude that microbiota variability is correlated to clinical conditions such as pancreatitis and premalignant or malignant lesions. With this in mind, by gathering more data, the microbial composition of PDAC patients could serve as a prognosis indicator: Alpha-proteobacteria, Proteobacteria, and Actinobacteria- rich patients showed longer overall-survival rates.

As a non-invasive screening tool, oral bacterial genera (in particular, *Haemophilus, Porphyromonas, Leptotrichia and Fusobacterium spp*.) need further insight, seeing that their abundance is altered in PDAC patients, along with anti-*P.gingivalis* and anti-*Aggregatibacter spp* antibodies. Therefore, analysis of oral microbiota and related serology may be a more specific indicator, which needs to be further studied and compared to current tools like CA 19.9 levels.

As a therapeutic tool, direct use of modified bacteria used as cargo for chemotherapy drugs (attenuated *S. typhimurium* and *E. coli*, *S. pyogenes* and *Lactobacillus*), or an indirect approach with bacteria- derived metabolites (namely, TMAO and 3-IAA) reported positive results: the latter would provide an easier use, as metabolites are easier to quantify and administer, with a reduced risk of excessive immune reactions in patients, which would pose a potential threat to their wellbeing. These approaches need extensive further research in order to reach standardized protocols, however they have the potential for a more precisely targeted therapy, surpassing current chemotherapy and leaving behind harmful side effects which come from current protocols.
